# Use of mHealth Technologies to Increase Sleep Quality among Older Adults: A Scoping Review

**DOI:** 10.3390/clockssleep6030034

**Published:** 2024-09-04

**Authors:** Giulia Grotto, Michela Martinello, Alessandra Buja

**Affiliations:** 1 Department of Pharmaceutical and Pharmacological Sciences, University of Padua, Via Marzolo, 5-35131 Padua, Italy; 2 Institute of Anesthesia and Intensive Care Unit, University Hospital of Padua, Via Vincenzo Gallucci, 13-35121 Padua, Italy; michela.martinello.2@studenti.unipd.it; 3 Department of Cardiological, Thoracic and Vascular Sciences, and Public Health, University of Padua, Via Loredan, 18-35127 Padua, Italy; alessandra.buja@unipd.it

**Keywords:** mHealth, insomnia, sleep hygiene, older adults

## Abstract

Sleep disorders increase with age and are known risk factors for several mental and physical diseases. They also significantly contribute to a lower quality of life. Nonpharmaceutical approaches, such as cognitive behavioral therapy for insomnia, sleep hygiene advice, relaxation exercises, and physical activity programs, can be delivered directly to patients via mHealth technologies, thereby increasing the accessibility of such interventions and reducing health care-related costs. This scoping review aims to evaluate the effectiveness of mHealth interventions for improving sleep quality among older adults. Published studies in the last 10 years (2013–2023) were identified by searching electronic medical databases (PubMed, PsycINFO, CINAHL, and Scopus) in July 2023 and were independently reviewed by two different authors. The analysis of the data was performed in 2023. The research retrieved 693 records; after duplicates were removed, 524 articles were screened based on their title and abstract, and 28 of them were assessed in full text. A total of 23 studies were excluded because they did not meet the inclusion criteria in terms of population age (60 years or over) or type of intervention (mHealth-based) or because they addressed secondary insomnia. A total of five studies were included in this review, and all of them reported improvements in subjective sleep quality after the application of the mHealth interventions. Two studies also conducted objective assessments of sleep outcomes using actigraphy, reporting improvements only in some of the variables considered. Despite the limited number of available studies, these results are promising and encourage further research.

## 1. Introduction

Worldwide, the population of older adults is increasing and is expected to reach two billion by the year 2050 [1]. As people age, their sleep patterns tend to change [2], and the likelihood of experiencing sleep issues rises after the age of 65 [3,4,5]. About half of all older individuals struggle with sleep problems [6,7,8], including insomnia in 30% of the cases and sleep apnea in 20% [4,9]. Inadequate sleep health can be identified by insufficient or excessive sleep time, low sleep quality, sleep interruptions, inconsistent sleep schedules, and/or daytime impairment [10]. Symptoms of insomnia, like trouble falling or staying asleep, waking up early in the morning, and feeling sleepy during the day, are the most prevalent sleep issues among older adults; these symptoms impact 30–50% of all individuals [11]. Sleep disorders can have a negative impact on quality of life and are commonly linked to symptoms of mental and physical health problems [12,13,14,15,16]. For instance, older adults with sleep disorders have a higher susceptibility to cardiovascular [17,18] and metabolic conditions [19,20]. Moreover, insomnia has been linked to a higher risk of developing all-cause dementia [21], increased mortality, and psychiatric comorbidities in older adults with or without dementia [21] as well as being a potential factor in the development of depression [22]. In addition, sleep problems in older adults affect general functioning and can lead to fatigue, daytime sleepiness, and napping [23,24].

Quality of sleep shifts with age, resulting in changes in duration and consolidation as well as in increased sleep fragmentation, earlier wake-up times, and decreased slow wave sleep [25]. The sleep issues experienced by older individuals are usually the result of multiple factors, including primary sleep disorders like obstructive sleep apnea, periodic limb movements during sleep, and restless legs syndrome, or they may stem from underlying medical conditions, psychiatric disorders, medication effects, or social influences [25]. Nonetheless, the multifaceted nature of sleep quality hinders its empirical evaluation. Sleep quality is typically evaluated by utilizing a range of methods, such as subjective assessments like the Consensus Sleep Diary and the Pittsburgh Sleep Quality Index as well as objective measures like polysomnography and actigraphy [26].

Just half of all reported sleep issues can be attributed to a diagnosed sleep disorder [27]. Nevertheless, the majority of sleep health interventions focus on clinical insomnia, with fewer interventions designed for adults experiencing poor sleep without a diagnosed sleep disorder [28]. Consequently, individuals experiencing poor sleep health may not be utilizing available resources to enhance their sleep quality. Both pharmaceutical and non-pharmaceutical approaches are available for treating sleep issues. Although benzodiazepines, non-benzodiazepines, and antidepressants are frequently prescribed to older adults [29], they can result in side effects and may not be consistently safe or successful in the long term [30,31,32]. In fact, the extended use of sedative-hypnotics is not recommended because it can lead to various negative side effects, including falls, nausea, confusion, dizziness, headaches, daytime drowsiness, abuse and dependence, memory impairment, or rebound insomnia [33,34,35]. A variety of non-pharmacological interventions are now more widely accessible. Non-pharmaceutical interventions such as cognitive behavioral therapy for insomnia (CBT-I), sleep hygiene recommendations, relaxation techniques, and exercise have been shown to be effective [9,28,36,37,38,39,40,41]. In particular, CBT-I is the preferred first-line therapy for insomnia [42,43], and studies suggest it can enhance sleep quality in individuals without a diagnosed sleep disorder [28].

Although most health promotion and disease prevention interventions for older adults are conducted in a traditional mode [44], such as face-to-face counseling, there is a growing trend towards using information and telecommunication technologies in healthcare; over the last ten years, mobile health (mHealth) has rapidly advanced and demonstrated significant potential in transforming people’s health habits [45]. Specifically, mHealth technologies offer distinct features that are not commonly seen in traditional care, like medical advice delivered through personalized text messages or customized phone reminders that promote specific health actions, such as exercising or medication adherence [46]. In particular, mHealth utilizes various mobile technologies like smartphones, electronic activity monitors, and wireless technology to provide healthcare services [47]. Technology has become increasingly common in high-income countries in recent years, with most individuals aged 65 or older owning a cell phone and being comfortable using mobile devices in their everyday activities [48,49].

The application (app) systems found on digital devices have the potential to be customized to promote sleep hygiene, increase sleep quality, and facilitate nonpharmacological treatment for insomnia among the older adult population. mHealth has already been incorporated into health promotion activities for older adults; for instance, in a previous study, the effectiveness of mHealth strategies, such as goal setting, self-monitoring, motivational messages, activity reminders, and phone coaching for physical activity in older adults, was tested, proving the feasibility of incorporating a combination of these mHealth strategies in interventions for older adults [50]. Developing mobile apps to assist with insomnia treatment and promote sleep hygiene could lower clinics’ costs and improve patient access to clinical assistance. In fact, digital technologies permit the remote delivery of health promotion interventions, enabling older adults to access these programs while away from their homes. Consequently, the flow and delivery of health care services become more efficient, widespread, and less expensive. Additionally, mHealth interventions have the capacity to offer broad, extensive, and easily accessible delivery of cost-effective behavior change interventions when compared to in-person interventions [51]. mHealth interventions can reduce symptom severity in adults with diagnosed sleep disorders or secondary insomnia [52], yet there is a lack of studies on the effectiveness of mHealth sleep interventions in older adults with primary insomnia.

This scoping review aims to examine recent literature on interventions utilizing mHealth technologies to improve sleep quality and treat insomnia in older adults and to assess these technologies’ effectiveness.

## 2. Materials and Methods

### 2.1. Search Strategy and Data Extraction

The review methods were pre-established according to the Preferred Reporting Items for Systematic Reviews and Meta-Analysis (PRISMA) guidelines [53]. For the present study, the PubMed, PsycINFO, CINAHL, and Scopus databases were systematically searched in July 2023. The search strategies were developed by combining the keywords of the three main axes of the research (“older adults”, “Insomnia”, and “mHealth”) and respective synonyms using Boolean operators. For the PubMed database, a combination of MeSH terms and text words was used. The search strategies are provided in Appendix A.

Two authors (GG and MM) independently assessed the eligibility of the retrieved studies in a two-stage process: first, a screening based on titles and abstracts, followed by a screening of the full text. Reviewers resolved disagreements by reaching a consensus or seeking input from another author (AB). The authors searched the reference lists of the included studies to find more relevant articles.

An Excel-based data extraction form was created to address the research question. Data extracted from the studies included: (1) primary author, year, setting, and study design; (2) study objective; (3) sample size and population characteristics; (4) intervention details; (5) measure of interest; (6) results; and (7) conclusions. The characteristics of the included studies were reported through descriptive analysis (Table 1 and Table 2).

### 2.2. Inclusion Criteria

Studies meeting the following criteria were considered for inclusion in the review:

- focused on improving sleep quality or contrasting primary insomnia;
- implemented an mHealth-based intervention;
- included individuals who were 60 years old and older;
- were interventional studies (i.e., guidelines, reviews, letters, and editorials were excluded);
- were published in the last 10 years (2013–2023); and
- were written in English.

Studies aimed at treating insomnia secondary to other clinical issues (i.e., depression, generalized anxiety disorder, and osteoarthritis-related insomnia) were excluded.

## 3. Results

### 3.1. Identified Studies

**Figure 1:**
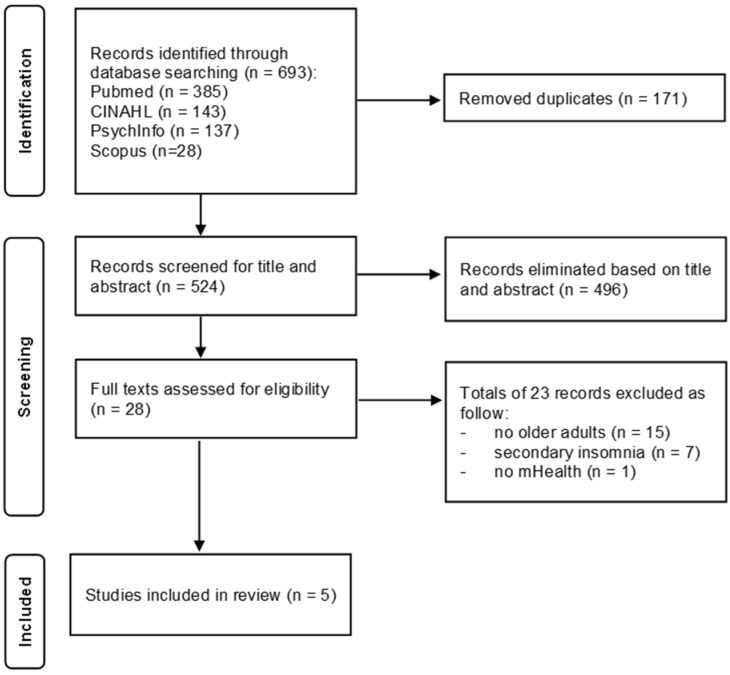
The Preferred Reporting Items for Systematic Reviews and Meta-Analyses (PRISMA) diagram of the article search.

The initial search yielded 693 citations from the electronic databases. After removing duplicates, 524 titles and abstracts were screened, and 28 were assessed in full text for eligibility. Among them, 23 studies were excluded for the following reasons: they were not related to the target population (fifteen studies), they concerned insomnia secondary to other diseases (seven studies), or they did not include an mHealth intervention (one study). Ultimately, five studies met the inclusion criteria. No additional studies were included based on the manual search of the reference lists. The literature search is shown in detail in Figure 1. The characteristics and results of the included studies are shown in Table 1 and Table 2, respectively.

### 3.2. Characteristics of the Included Studies

The five included studies were different in terms of the study design: one randomized controlled trial (RCT) [57], one pre-post test study with a control group [58], two pre-post test studies with no control group [55,56], and one case report [54]. Additionally, the included studies were conducted in different parts of the world: two studies were conducted in Asia (Taiwan [54] and Korea [55]), one was conducted in the United States [54], one was conducted in Australia [56], and one was conducted in Iran [58].

The main outcome of interest was the Pittsburgh Sleep Quality Index (PSQI) [59], which was used in four of the five studies [55,56,57,58]. Two of these studies also performed actigraphy (measure of sleep through the Actiwatch) and implemented the use of a sleep diary [56,57]; in addition, one study also evaluated dysfunctional sleep beliefs, using the Dysfunctional Beliefs and Attitudes about Sleep scale (DBAS-16) [56,60]. The fifth study evaluated subjective sleep satisfaction through a Likert scale (from 0 = “very bad” to 3 = “very good”) [54]. Sleep quality measurements were taken in all studies at least twice, before and after the intervention. In the controlled studies [57,58], the differences in sleep quality before and after the intervention were compared between the intervention and control groups.

The five studies included a total of 147 community-dwelling older adults, the majority of whom were women (*n* = 112). The mean age of the participants was 69 years.

Three of the interventions applied CBT-I through mHealth technologies (tablets and smartphones) [54,55,56], while the other two aimed to improve sleep quality through the promotion of adequate physical activity [54] and through self-care training programs via mHealth technologies that promote healthier nutrition, exercise, management of common chronic disease, and mental health [58]. The duration of the interventions ranged from one [55] to twenty-four weeks [57].

In relation to different mHealth strategies, the interventions were delivered through devices such as smartphones [55,58], tablets [54], or smartwatches [57]. Specially designed apps [54,55], social network chats [58], or online websites [56] were used. Content was delivered through multimedia files (videos or clips) [58], messages [54,55,56,57], or infographics [54]. Typically, educational interventions delivered information through an organization in modules. Examples of modules included the following: sleep hygiene, maladaptive cognitive processes and behaviors related to sleep, sleep restriction, stimulus control, cognitive therapy, relaxation techniques, healthy nutrition, healthy lifestyle and purposeful activity, and mental health [54,55,56,58].

### 3.3. Study Results

All five studies reported improvement in subjective sleep quality after the application of the mHealth interventions; in particular, the RCT showed that the intervention significantly improved the PSQI scores at 16 weeks (mean difference = 1.1, *p* = 0.046) and postintervention (24 weeks) (mean difference = 2.3, *p* = 0.02) after controlling for baseline values [57]. The three pre-post test studies implementing CBT-I showed that after the intervention, the overall mean score of the PQSI decreased from 7.44 to 5.77 (*p* = 0.001) [58], from 8.00 to 5.11 (*p* = 0.006) [55], and by 1.34–2.49 points [56], indicating improved sleep quality. Finally, the case report showed that after providing CBT-I, the subjective sleep satisfaction ratings gradually improved from 1.5 to 2.0 (on a 0–3 Likert scale) after five weeks, resulting in the successful discontinuation of all the hypnotics [54].

Two studies performed an objective evaluation of sleep outcomes using actigraphy [56,57]; the physical activity-based intervention significantly improved the actigraphy-measured nighttime sleep duration (mean difference = 10.8 min, *p* = 0.02) and the sleep efficiency (mean difference = 3.6%, *p* = 0.02) on the intervention group (no differences in objective sleep onset latency and wake after sleep onset) [57]; the CBT-I intervention showed that complaining poor sleepers objectively decreased wake after sleep onset by almost 12 min, along with a decrease in total sleep time of over half an hour [56]. No objective differences were found for the other groups (non-complaining poor sleepers, complaining good sleepers, and non-complaining good sleepers); nor were any objective differences found regarding the number of nocturnal awakenings and sleep onset latency [56].

This study also assessed how participants subjectively perceived the variables mentioned. The results showed the following: no difference in total sleep time or in sleep onset latency, a decrease of wake after sleep onset only among the complaining poor sleepers, and a decrease in the number of awakenings among the complaining good sleepers [56]. Dysfunctional sleep beliefs decreased in all three groups experiencing sleep issues (complaining good sleepers, complaining poor sleepers, and non-complaining poor sleepers) following the intervention [56].

## 4. Discussion

All five studies included in this scoping review reported that mHealth interventions were effective in improving the subjective sleep quality of older adults, as evaluated using the PSQI.

Regarding the objective assessment of other sleep outcomes, the two studies that utilized actigraphy demonstrated improvements in different variables: the physical activity-based intervention resulted in an increase in total sleep time and sleep efficacy (which is defined as the ratio of total nocturnal sleep time to total time in bed) [57]; and the CBT-I intervention showed a decrease in wake after sleep onset but also led to a reduction in total sleep time among the complaining poor sleepers [56]. The unexpected decrease in total sleep duration after CBT-I reported by Kutzer et al. might have been due to sleep restriction, which is a component of the CBT-I program [56].

Kutzer et al. also examined the impact of CBT-I on dysfunctional sleep beliefs and found that they were significantly reduced in complaining good and poor sleepers as well as in non-complaining poor sleepers. This suggests that CBT-I may reduce dysfunctional sleep beliefs, which in turn helps alleviate symptoms of insomnia [56]. As dysfunctional sleep beliefs decreased in all sleep groups except for normal sleepers, this suggests that sleep issues in these older adults may have a significant cognitive aspect that may not be resolved solely by providing sleep medication [56].

Assessing sleep can be challenging and may involve measurement issues. Subjective sleep quality is usually evaluated through the use of a sleep diary. This can be a valuable tool for assessing insomnia and monitoring sleep habits and patterns, and it is preferable to adopt a standardized version, such as the Consensus Sleep Diary [61], that will facilitate comparisons across studies. Subjective sleep evaluations should always be accompanied by an objective evaluation, especially among older adults [54]. In fact, fragmented sleep is a normal part of aging and more common in older adults, and it might be difficult for participants to recall all awakening events during sleep [54]. The simultaneous use of actigraphy (i.e., wearable devices with an embedded accelerometer) can supplement this and other missing information.

Actigraphy sleep assessment is utilized for the objective assessment of various sleep parameters, such as sleep onset latency, wake after sleep onset, total sleep time, number of nighttime awakenings, and sleep efficacy. However, sleep efficacy may not be a reliable indicator in sleep restriction programs as it is calculated with time in bed as the denominator, which can create a methodological problem in sleep intervention research where sleep restriction therapy is employed. The enhancement of sleep efficiency may occur as a result of participants complying with guidelines to decrease time spent in bed, not as a result of the intervention’s effects [56]. Also, actigraphy’s precision in measuring sleep onset latency is inferior to that of polysomnography. However, actigraphy offers the advantage of monitoring objective sleep in a realistic context [56].

In recent times, there has been an increase in the adoption of wearable sleep-tracking devices, both in scientific research on sleep and among consumers. These tools present a possibility for constant, discreet, and widespread tracking of sleep habits in the person’s regular sleep setting. Consumer-grade sleep-tracking tools can differ greatly in their methods, hardware components, software features, and overall performance [62]. Given this, the criteria for assessing the effectiveness of wearable sleep trackers have been established, and current data indicate that consumer-level devices outperform traditional actigraphy in measuring sleep compared to polysomnography, with a steady progression showing enhanced accuracy over time [63]. Nevertheless, there are clear limitations for both consumer-level devices and traditional research/clinical actigraphy, including the misidentification of wakefulness while asleep, challenges in monitoring sleep outside the primary nighttime period, technical interferences, and the unclear translation of the performance of individuals with certain characteristics or person-specific factors (like skin color or obesity) that may impact sensor effectiveness adversely [63].

Even though some consumer-grade sleep-tracking devices outperform traditional actigraphy, their suitable application for research or clinical use is highly complex and poses challenges. Devices intended for consumers are equipped with optimized user interfaces and experiences, as well as functionalities that enhance ease of use. Nevertheless, since the main goal is to monitor sleep for consumer information, there might be undisclosed algorithms, inaccessible raw data, privacy and security issues, lack of control over software updates, unknown consistency and reliability of hardware components, and uncertain long-term availability of devices and models, all of which pose notable limitations for research and clinical purposes [63].

The five analyzed studies utilized two main approaches to improve sleep quality: the use of CBT-I and the promotion of healthier behaviors, such as physical activity and other lifestyle habits that positively impact sleep health.

Regarding the CBT-I, numerous previous studies have revealed that traditionally administered (i.e., in-person) CBT-I alone was more effective than pharmacotherapy alone or combined treatment after treatment administration and discontinuation [33,34,64,65,66,67]. However, traditionally delivered CBT-I has not been widely adopted, possibly because of the limited availability of CBT-I services [68]. Confronted with this constraint, prior studies have investigated offering CBT-I through self-help methods like booklets, videos, tapes, or online platforms [69]; in this scenario, the use of mHealth technologies could help to overcome this limitation in the delivery of CBT-I. Also, traditional CBT-I’s effectiveness could be compromised by problems with adherence, such as drop-out, premature termination, irregular session attendance, and the failure to complete homework [70]. Traditionally, patients engage in a variety of behavioral self-management activities to make lifestyle changes that will help reduce sleep disturbances, and they usually maintain a daily sleep diary and record their activities on paper to support the behavioral intervention. Because of these typical characteristics of CBT-I, older adults face difficulties in understanding and adhering to the treatment instructions due to their declining perceptual and cognitive abilities [54]. Implementing CBT-I through a mobile app for self-management among older adults could be a promising solution that addresses adherence issues because this approach could provide older adults with extra guidance on how to effectively complete the necessary tasks [55]. For instance, apps can send timely automatic reminders to prompt patients to perform prescribed time-sensitive behavioral assignments, or they can provide multimedia content designed to promote relaxation to guide on-demand practice [54]. In addition, the use of a digital sleep diary allows therapists to monitor sleep patterns/trends and offers instant updates on the patient’s advancement. Also, using a personal mobile device to record diary entries can help users avoid extra memory strain and protect against data loss [54].

Sleep restriction therapy, a component of the CBT-I protocol, restricts the use of CBT-I whether it is delivered in person or through mHealth. In fact, sleep restriction might lead to increased daytime sleepiness. Therefore, individuals with conditions that can be negatively impacted by sleepiness, such as epilepsy, sleep-disordered breathing, bipolar disorder, or schizophrenia, are not recommended for participation in this treatment [56]. In addition, older adults may be more prone to falls due to decreased cognitive function caused by lack of sleep [56]. Hence, seniors with epilepsy, a history of obstructive sleep apnea, bipolar disorder, schizophrenia, or a high risk of falling should avoid participating in sleep restriction programs.

On the other hand, older adults may experience problems using CBT-I delivered through mHealth technologies due to perceptual impairments. In the case report by Chen et al., visual impairments were compensated for by using a large tablet, although this could compromise the portability of smartphones and consequently the function of the reminder systems [54]. Additionally, the fragmentation of sleep typical in older adults might limit the capability to recall all awakening events, but this problem could be overcome by the adoption of a wrist sleep sensor to automatically collect objective information, such as cumulative duration of waking after sleep onset [54].

Earlier investigations have centered on evaluating the efficiency, viability, and user-friendliness of CBT-I and other mobile health applications for older adults [71,72,73]. Regarding age-related challenges in usability assessments, it was observed that a considerable number of high-level usability problems when using mHealth apps were more closely associated with motivational hurdles, such as limited computer skills and self-doubt in app usage abilities, rather than cognitive and perceptual barriers like memory and visual acuity [73]. Therefore, it is crucial to introduce further educational programs to help older adults become more comfortable with mobile devices and apps, leading to a positive impact on their perceived usability and self-efficacy in mHealth technologies [55].

Concerning the role of physical activity in fostering sleep quality, earlier research has indicated that older individuals who participate in higher levels of physical activity often have fewer insomnia symptoms and experience improved sleep quality [74,75,76,77,78].

The included study by Li et al. supported this hypothesis and showed that self-reported sleep quality started to improve at 16 weeks after the beginning of the intervention and that the enhancement in sleep variables measured objectively began after 24 weeks [57]. These results imply that the impact of physical activity programs on sleep patterns might not manifest immediately but could show up weeks later, aligning with conclusions drawn in an earlier meta-analysis [77].

Vizeshfar et al. implemented a self-care program for older adults, which is described as a holistic approach impacting individuals’ lifestyle through physical activity, nutrition, sleep, and mental health [79]. The authors suggested that the improvement in sleep quality and overall health they found in their sample was prompted by the emphasized physical activity and attention to mental health [58]. Changing people’s physical activity behaviors or lifestyles is difficult, even though the benefits of exercise and other health behaviors are widely recognized. Examples of obstacles that often prevent older adults from engaging in physical activity include poor health, lack of interest, fear of falling, pressure from group exercise, inconvenience, and cost [80]. In addition, as people age, their levels of physical activity tend to decrease along with a decline in sleep health. This indicates that older adults should be a key focus for interventions that combat the negative effects of being inactive and having poor sleep hygiene [81].

Although sleep interventions often suggest incorporating “regular exercise” into sleep hygiene practices [82], there is a lack of specific strategies and behavior change techniques provided to enhance physical activity [69]. This holds true even though compelling evidence indicates that certain behavior change techniques (such as self-monitoring and action planning) are necessary to successfully alter each desired behavior in various health behavior change programs [83]. As a result, efforts to improve sleep may not take full advantage of the potential benefits that could come from boosting physical activity levels. In this regard, mHealth easily allows the implementation of strategies, such as goal setting, self-monitoring, motivational quotes, and activity reminders, which have shown promise for changing individuals’ health behaviors, including physical activity [45,50,84].

In general, educational interventions focused on self-care can enhance overall health. Sleep quality, as a crucial aspect of general health, could also improve as a result of these interventions [58]. The most crucial self-care behaviors for promoting health include maintaining a healthy lifestyle to prevent chronic diseases, adopting healthy eating habits, having control over decision-making, feeling content with one’s life, managing stress effectively, and addressing sleep disorders [58]. As we age, health-promoting behaviors become increasingly important and, among the factors influencing health, adopting healthy self-care habits is considered the most essential way to prevent diseases, especially chronic conditions [58]. Therefore, prioritizing health-promoting self-care behaviors should be seen as the key strategy for maintaining and enhancing health.

Standardized interventions may have a limitation in that they offer the same information to all participants without considering the specific characteristics of sleep issues and the individual needs of each person. Individual differences in sleep problems should be considered when using a mobile application for self-help CBT-I without the guidance of a therapist [55]. To enhance the effectiveness of treatment, it is advisable to conduct a pre-treatment assessment session aimed at identifying the factors that lead to or maintain sleep problems; this can be done through a clinical interview, filling out a sleep history questionnaire, or keeping a sleep diary before beginning the treatment program [55]. It would be appropriate for the interventions to be supervised by healthcare professionals who are familiar with the participant and their sleep issues, providing personalized feedback and monitoring progress, even remotely [58]. For example, in Chen et al.’s study, therapists could track sleep patterns or trends documented in the digital sleep diary and offer feedback on patients’ progress [54]. Then, therapists or technical assistants managed and updated the CBT-I-related assignments for their individual patients on a private website [54]. The assignments on the patient’s device were updated through an automated data synchronization mechanism that refreshed data between the client and server every hour and allowed the therapist to remotely monitor the patient’s condition [54].

In conclusion, mHealth interventions have shown promise in enhancing sleep health in older adults, reaching a wide audience, being easily accessible and cost-effective, offering flexibility to patients, and requiring less time from specialists. In addition, incorporating special tools like animations, multimedia content, dynamic feedback, interactivity, and social networking can improve compliance and increase the perceived value of the intervention [69].

One significant limitation of this review is the small number of studies and the limited sample size found in the literature, leading to uncertainty in the conclusions. Also, the included studies exhibit heterogeneity in research design and types of intervention. However, the consistency of the results in the analyzed publications is promising and encourages the development of more studies assessing the effectiveness of interventions aimed at improving sleep quality among older adults through mHealth intervention programs.

## 5. Conclusions

Mobile technology has been used extensively worldwide in recent decades, and mHealth has developed in parallel with it. This method offers the advantages of enhanced availability of medical services in all locations, speedy access, and simplicity. The use of mHealth and telemedicine has become increasingly important for healthcare providers, particularly in light of the COVID-19 pandemic, with a heightened significance for older adults who are more vulnerable and have limited mobility. Although the number of studies is still limited and further research is needed, all five studies included in this review showed promising results regarding the application of mHealth interventions aimed at improving subjective sleep quality among older adults. These results can support the use of mHealth interventions to improve insomnia symptoms in older adults who cannot access traditional interventions. Given that the two studies that analyzed sleep quality through objective methods yielded mixed results in some of the considered variables, it is desirable for future studies to analyze objective sleep quality (e.g., through actigraphy) in addition to subjective sleep quality. Furthermore, future research should focus on studies with a less biased design, such as RCTs, to provide more robust evidence.

## Figures and Tables

**Table 1:** Characteristics of the included studies.

Main Author, Year, Setting. Study Design	Aim	Sample Size and Characteristics of the Population	Intervention	Measures of Interest
Chen et al., 2016 [54], Taiwan. Case Report	To examine the usage, benefits, and restrictions of mobile app-facilitated cognitive behavioral therapy for insomnia (CBT-I) in an older female individual.	An insomnia-afflicted 64-year-old Chinese woman	A sleep specialist delivered six sessions of CBT-I through a mobile app. Duration of the intervention: 5 weeks.	Likert scale assessments of subjective sleep satisfaction (from 0 = “very bad” to 3 = “very good”)
Chung et al., 2020 [55], Republic of South Korea. Pre-post test study	Analyzing improvements in subjective sleep quality after a 1-week intervention period using a mobile app for self-managing insomnia	9 community-dwelling Korean women. Mean age 71.56 years, SD 4.36 years	A mobile app for CBT-I providing a self-management intervention focusing on educating users about sleep hygiene, implementing sleep restriction, practicing stimulus control, and engaging in cognitive therapy. Duration of the intervention: 1 week.	Pittsburgh Sleep Quality Index (PSQI)
Kutzer et al., 2023 [56], Australia. Single arm non-randomized trial with a pre/post-test design	To examine the impact of uncoupled sleep (disconnection between sleep pattern and sleep complaint) on dysfunctional sleep beliefs and sleep outcomes in community-dwelling older adults following digitally delivered CBT-I and to assess how these groups respond to CBT-I	62 community-dwelling older adults aged 60–80 years. 55 females, mean age 66 years, SD 3.82 years	Phase 1: online questionnaires assessing sleep oucomes, sleep quality (PSQI), and dysfunctional sleep beliefs (DBAS-16); objective and subjective sleep measures evaluated using an actigraph and a sleep diary over 96 h. Based on their sleep complaint status, participants were grouped into four sleep categories: non-complaining good sleepers, complaining good sleepers, non-complaining poor sleepers, and complaining poor sleepers. Phase 2: self-guided online CBT-I intervention	Objective and subjective sleep onset latency, wake after sleep onset, total sleep time, and number of nighttime awakenings; PSQI, DBAS-16
Li et al., 2022 [57], United States. Randomized controlled trial	To assess the impact of a 24-week mHealth personalized intervention on physical activity and sleep issues	21 community-dwelling older adults with self-reported poor sleep (intervention: *n* = 11, control: *n* = 10). Mean age: 73.3 years, SD 6.6;	24 weeks mHealth-facilitated, personalized intervention on physical activity and sleep which included: (1) mHealth technology learning sessions; (2) one in-person training session with the prescription of a personalized physical activity plan; (3) mHealth strategies for promoting physical activity (self-monitoring of levels of physical activity on the smartwatch, reminders etc.); (4) financial incentives to complete the prescribed physical activity (5$/week); and (5) additional support for mHealth technology	Objective sleep onset latency, wake after sleep onset, total sleep time, and sleep efficacy, sleep diary, and PSQI
Vizeshfar et al., 2021 [58], Iran. Controlled pre-post test study	To assess the impact of a self-care smartphone training program on the overall health, nutritional well-being, and sleep quality of older adults.	54 healthy and independent members of a day-center for older adults. Mean age 69.80, SD 11.60	21 sessions of self-care training (on the themes of healthy nutrition, exercise, management of common chronic diseases, mental health, and sleep problems) were prepared in the form of videos and sent to the participants through WhatsApp (one per day for 21 days). General Health Questionnaire, Mini Nutrition Assessment, and the PSQI were completed initially and at one month follow-up after the end of the program	PSQI

**Table 2:** Results and conclusions of the included studies.

Main Author, Year, Setting. Study Design	Results	Conclusions
Chen et al., 2016 [54], Taiwan. Case Report	Subjective sleep satisfaction ratings gradually improved: first week, 1.5 (SD = 0.7); third week, 2.0 (SD = 1.0); and fifth week, 2.0 (SD = 0.0). Additionally, after CBT-I, all hypnotics were successfully discontinued and a good sleep quality was maintained.	CBT-I delivered through mobile apps could improve sleep quality among the older adults.
Chung et al., 2020 [55], Republic of Korea. Pre-post test study	The subjective evaluation of sleep quality differed between preintervention (mean 8.00, SD 2.50) and postintervention (mean 5.11, SD 1.36); the use of the app significantly improved sleep quality in the older adults after one week (3.74, *p* = 0.006).	Subjective sleep quality improved after the 1-week self-help intervention period.
Kutzer et al., 2023 [56], Australia. Single arm non-randomized trial with a pre/post-test design	Objective measures—sleep onset latency and n. of awakenings: no difference; total sleep time: decreased by over half an hour in complaining poor sleepers; wake after sleep onset: decreased almost 12 min in the complaining poor sleepers, no changes in the other groups; Subjective measures—sleep onset latency and total sleep time: no difference; wake after sleep onset: decreased in the complaining poor sleepers; number of awakenings: lower for the complaining good sleepers; dysfunctional sleep beliefs: lower for complaining good sleepers, complaining poor sleepers, and non-complaining poor sleepers; sleep quality: lower PSQI scores for all groups	Objective and subjective wake after sleep onset decreased in the complaining poor sleepers group. The number of subjective nocturnal awakenings decreased in complaining good sleepers. Total objective sleep time decreased in complaining poor sleepers (likely due to sleep restriction performed in CBT-I). Dysfunctional sleep beliefs decreased in all groups apart from normal sleepers. Self-reported sleep quality improved in all groups. CBT-I outcomes might be influenced by the level of dysfunctional sleep beliefs
Li et al., 2022 [57], United States. Randomized controlled trial	Post-intervention results showed a significant improvement in actigraphy-measured nighttime sleep duration (MD = 10.8 min, *p* = 0.02) and sleep efficiency (MD = 3.6%, *p* = 0.02) in the intervention group compared to the control group. After adjusting for baseline values, self-reported sleep quality in the intervention group saw enhancements at both 16 weeks (MD = 1.1, *p* = 0.046) and postintervention (MD = 2.3, *p* = 0.02)	Mobile health interventions for physical activity may enhance both physical activity levels and sleep quality in older adults
Vizeshfar et al., 2021 [58], Iran. Controlled pre-post test study	The PSQI showed that the overall mean score decreased from 7.44 to 5.77 post-intervention (*p* = 0.001). Before and after the intervention, there were no significant differences observed in three of the 7 subscales (mental quality of sleep, drug use, and sleep disorders)	The use of smartphones for delivering training on self-care habits can play a significant role in enhancing the health of older individuals

## Data Availability

No new data were created or analyzed in this study.

## Appendix A

PubMed search string

(“old people”[Title/Abstract] OR “old adult*”[Title/Abstract] OR “old age”[Title/Abstract] OR “older people”[Title/Abstract] OR “older adult*”[Title/Abstract] OR “older age”[Title/Abstract] OR “geriatric*”[Title/Abstract] OR “elder*”[Title/Abstract] OR “senior*”[Title/Abstract] OR older person*[Title/Abstract] OR old person*[Title/Abstract] OR aging adult*[Title/Abstract] OR aging person*[Title/Abstract] OR “Aged”[Mesh]) AND (telemedicine[MeSH Terms] OR telemedicine[Title/Abstract] OR “Mobile Health”[Title/Abstract] OR “Telehealth”[Title/Abstract] OR “mHealth”[Title/Abstract] OR “eHealth”[Title/Abstract] OR “m-health”[Title/Abstract] OR “e-health”[Title/Abstract] OR “SMS”[Title/Abstract] OR “MMS”[Title/Abstract] OR “digital”[Title/Abstract] OR “smartphone”[Title/Abstract] OR “smartphones”[Title/Abstract] OR “Cell Phone”[Title/Abstract] OR “Cellular Phone”[Title/Abstract] OR “Telephone”[Title/Abstract] OR “Mobile Phone”[Title/Abstract] OR “iPhone” [Title/Abstract] OR “Cell Phones”[Title/Abstract] OR “Cellular Phones”[Title/Abstract] OR “Telephones”[Title/Abstract] OR “Mobile Phones”[Title/Abstract] OR “Message”[Title/Abstract] OR “Messages”[Title/Abstract] OR “Messaging”[Title/Abstract] OR “Text-Messaging”[Title/Abstract] OR “Texting”[Title/Abstract] OR “Email”[Title/Abstract] OR “E-Mail”[Title/Abstract] OR “Electronic Mail”[Title/Abstract] OR “Emails”[Title/Abstract] OR “E-Mails”[Title/Abstract] OR “Mobile Application”[Title/Abstract] OR “Mobile Applications”[Title/Abstract] OR “Mobile Apps”[Title/Abstract] OR “Mobile App”[Title/Abstract] OR “App”[Title/Abstract] OR “Smartphone Apps”[Title/Abstract] OR “Smartphone App”[Title/Abstract] OR “notification”[Title/Abstract] OR “notifications”[Title/Abstract] OR “reminder”[Title/Abstract] OR “alert”[Title/Abstract])AND (“Sleep Hygiene”[Mesh] OR “Sleep Hygiene”[Title/Abstract] OR “Sleep Quality”[Title/Abstract] OR “Sleep Duration”[Mesh] OR “Sleep Duration”[Title/Abstract] OR “Good Sleep”[Title/Abstract] OR “sleep disorder*”[Title/Abstract] OR “insomnia”[Title/Abstract]) Filters: in the last 10 years, English

PsycINFO and CINAHL search string, EBSCO database:

AB ((“old people” OR “old adult*” OR “old age” OR “older people” OR “older adult*” OR “older age” OR “geriatric*” OR “elder*” OR “senior*” OR older person* OR old person* OR aging adult* OR aging person* OR “Aged”))AND AB ((telemedicine OR telemedicine OR “Mobile Health” OR “Telehealth” OR “mHealth” OR “eHealth” OR “m-health” OR “e-health” OR “SMS” OR “MMS” OR “digital” OR “smartphone” OR “smartphones” OR “Cell Phone” OR “Cellular Phone” OR “Telephone” OR “Mobile Phone” OR “iPhone” OR “Cell Phones” OR “Cellular Phones” OR “Telephones” OR “Mobile Phones” OR “Message” OR “Messages” OR “Messaging” OR “Text-Messaging” OR “Texting” OR “Email” OR “E-Mail” OR “Electronic Mail” OR “Emails” OR “E-Mails” OR “Mobile Application” OR “Mobile Applications” OR “Mobile Apps” OR “Mobile App” OR “App” OR “Smartphone Apps” OR “Smartphone App” OR “notification” OR “notifications” OR “reminder” OR “alert”)) AND AB ((“Sleep Hygiene” OR “Sleep Hygiene” OR “Sleep Quality” OR “Sleep Duration” OR “Sleep Duration” OR “Good Sleep” OR “sleep disorder*” OR “insomnia”)). Limit to: English; published date: 1 July 2013–31 July 2023.

Scopus search string

(TITLE-ABS (old AND people)OR TITLE-ABS (old AND adult*)OR TITLE-ABS (old AND age)OR TITLE-ABS (older AND people)OR TITLE-ABS (older AND adult*)OR TITLE-ABS (older AND age)OR TITLE-ABS (geriatric*)OR TITLE-ABS (elder*)OR TITLE-ABS (senior*)OR TITLE-ABS (older AND person*)OR TITLE-ABS (old AND person*)OR TITLE-ABS (aging AND adult*)OR TITLE-ABS (aging AND person*)OR TITLE-ABS (ageing AND adult*)OR TITLE-ABS (ageing AND person*)OR TITLE-ABS (aged)) AND (TITLE-ABS (telemedicine)OR TITLE-ABS (mobile AND health)OR TITLE-ABS (tele AND health)OR TITLE-ABS (e-health)OR TITLE-ABS (m-health)OR TITLE-ABS (SMS)OR TITLE-ABS (MMS)TITLE-ABS (digital)OR TITLE-ABS (smartphone)TITLE-ABS (smartphones)OR TITLE-ABS (cell-phone)OR TITLE-ABS (cellular-phone)OR TITLE-ABS (telephone)OR TITLE-ABS (mobile-phone)OR TITLE-ABS (iPhone)OR TITLE-ABS (cell-phones)OR TITLE-ABS (cellular-phones)OR TITLE-ABS (telephones)OR TITLE-ABS (mobilephones)OR TITLE-ABS (message)OR TITLE-ABS (messages)OR TITLE-ABS (messaging)OR TITLE-ABS (text-messaging)OR TITLE-ABS (texting)OR TITLE-ABS (email)OR TITLE-ABS (e-mail)OR TITLE-ABS (electornic-mail)OR TITLE-ABS (emails)OR TITLE-ABS (e-mails)OR TITLE-ABS (mobile AND application)OR TITLE-ABS (mobile AND applications)OR TITLE-ABS (mobile AND apps)OR TITLE-ABS (mobile AND app)OR TITLE-ABS (app)OR TITLE-ABS (smartphone AND apps)OR TITLE-ABS (smartphone AND app)OR TITLE-ABS (notification)OR TITLE-ABS (notifications)OR TITLE-ABS (reminder)OR TITLE-ABS (alert)) AND (TITLE-ABS (Sleep AND Hygiene)OR TITLE-ABS (Sleep AND Quality)OR TITLE-ABS (Sleep AND Duration)OR TITLE-ABS (Good AND Sleep)OR TITLE-ABS (sleep AND disorder*)OR TITLE-ABS (insomnia)) AND PUBYEAR > 2012 AND PUBYEAR < 2024 AND (LIMIT-TO (LANGUAGE,”English”))
